# Mechanistic Analysis of Large Atomic Models of Molten Salt

**DOI:** 10.1002/advs.202522313

**Published:** 2026-03-10

**Authors:** Yuliang Guo, Xiaobo Sun, Xiaoli Xi, Zuoren Nie

**Affiliations:** ^1^ State Key Laboratory of Materials Low‐Carbon Recycling Beijing University of Technology Beijing China; ^2^ Collaborative Innovation Center of Capital Resource‐Recycling Material Technology College of Materials Science and Engineering Beijing University of Technology Beijing China

**Keywords:** density of states (DOS), electronic structure, machine‐learned interatomic potential (MLIP), molten salts

## Abstract

Machine‐learned interatomic potential (MLIP) has become a powerful tool to combine the accuracy of quantum mechanics with the efficiency of molecular dynamics in the era of artificial intelligence. However, a key open question persists: what physical mechanism is behind the atomic model that generates the MLIP and what physical information determines the final outputs? To address this problem, we use molten Na_2_WO_4_ as a representative system and fine‐tune a pretrained deep potential model (DPA2) with ab initio molecular dynamics data of Na_2_WO_4_. We find a strong correlation between the model's final output and the projected density of states (PDOS) in energy regions exhibiting high electron density and distinct local atomic environments. This result indicates that a well‐constructed neural network inherently captures the quantum‐mechanical information and its predictions represent meaningful physicochemical interactions rather than purely statistical patterns. Importantly, the mechanistic insights gained in this work—which links model's outputs to electronic structure descriptors— are general in nature. It provides an electronic‐structure‐informed metric for feature learning and a general strategy for building interpretable, transferable MLIPs across diverse material systems.

## Graphical abstract

This work uncovers the physical mechanism of large atomic models for molten salts by linking atomic contribution to electronic structure features. We demonstrate that energy predictions are physically determined by the local occupancy of frontier orbitals. This establishes a generalizable framework for building interpretable and transferable interatomic potentials across diverse material systems.

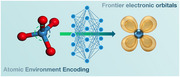

## 1 Introduction

Molten salts, owing to their high energy density, exceptional thermal stability, and robust chemical resistance, have become highly effective media for metal separation under extreme conditions [1, 2, 3, 4], especially are vital for fusion reactors, among other advanced applications [5, 6]. However, processing tungsten is notoriously difficult owing to its high melting point and hardness. Currently the most commonly used method for the controlled fabrication of high‐performance tungsten coatings is electrochemical methods utilizing molten Na_2_WO_4_ as electrolytes [6, 7, 8, 9, 10, 11, 12, 13].

Molecular dynamics (MD) simulation serves as a powerful tool for elucidating the microscopic mechanisms of molten salts [14, 15]. However, the lack of accurate and efficient interatomic potential models for the tungsten molten salt system poses a major bottleneck, restricting both fundamental insights and practical deployment. Machine‐learned interatomic potentials (MLIPs) [16, 17, 18], which combine the accuracy of first‐principles (DFT, density functional theory) calculations with the efficiency of classical MD, have recently driven major advances in materials modeling [19, 20, 21, 22, 23, 24, 25, 26]. MLIPs include models such as M3GNet [16] and CHGNet [17], which use graph neural network [27, 28, 29, 30, 31] have shown excellent performance [18, 21, 23, 32, 33, 34, 35, 36, 37, 38, 39, 40, 41, 42, 43]. These advancements underline the effectiveness of neural network‐based approaches in accurately modeling atomic interactions. Among these, large‐scale atomic models such as DPA2 have demonstrated excellent generalizability and predictive accuracy through multi‐task learning strategies [44]. A key strength of these models lies in their pretraining and fine‐tuning paradigm: an initial universal model is trained on a broad, diverse dataset to learn transferable representations, which are then refined through task‐specific fine‐tuning.

**FIGURE 1:**
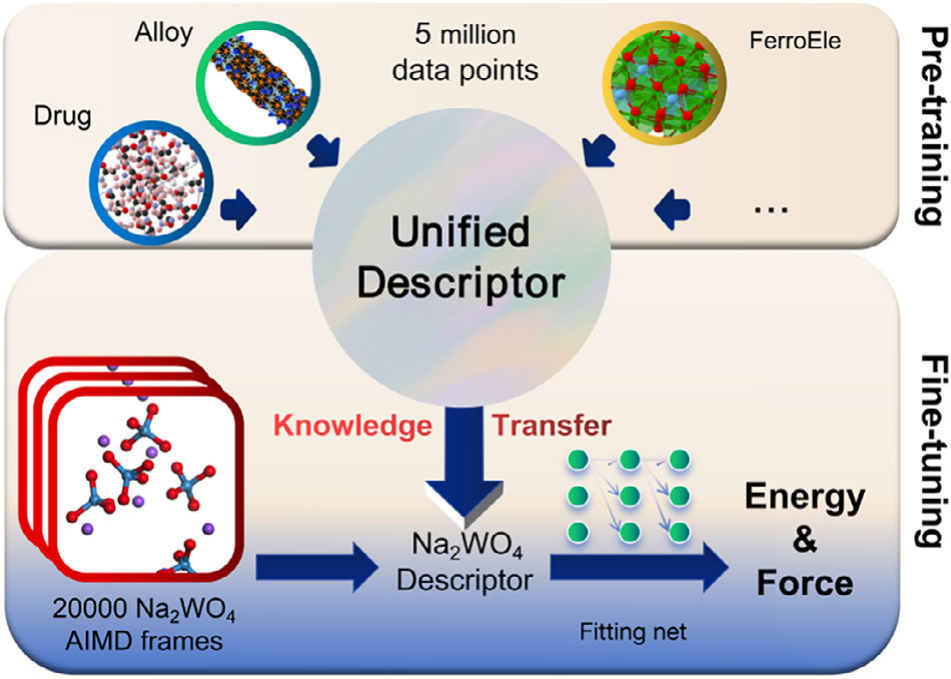
Using AIMD data of the Na_2_WO_4_ molten salt system to fine‐tune the pre‐trained DPA2 model.

Despite the widespread application of deep potential models across various fields, their specific use for complex ionic systems and the physicochemical implications of their atomic energy predictions currently suffers from a lack of sufficient exploration and clear understanding. As shown in Figure 1, we fine‐tuned a pretrained, high‐capacity DPA2 model using ab into molecular dynamics (AIMD) trajectories of molten Na_2_WO_4_ and systematically compare its performance with that of a model trained from scratch using the same architecture. Beyond evaluating the effectiveness of this transfer learning approach, we reveal a strong correlation between the model‐predicted atomic energies and the electronic structure, particularly with the projected density of states (PDOS) in energy regions exhibiting localized electron density and distinct atomic environments. This finding indicates that the model learns meaningful physical relationships between structure and energy, beyond superficial statistical correlations and offers new perspectives for both the development of interpretable machine‐learned potentials and a deeper understanding of DFT total energies.

In the following sections, we first validate the effectiveness of the pretrained model. To further elucidate its underlying rationale, we then perform DOS (density of stat) analysis on the molecular structures, demonstrating that the atomic contributions from the model outputs carry physical information.

## 2 Method

First, we used CP2K (v2023.1) [45] to generate training data, then model training was performed in the framework of DeepMD‐kit (v3.0.1) [46, 47], and finally subsequent molecular dynamics simulations were carried out using LAMMPS (v29Aug2024) [48]. Projected density of states (PDOS) analyses were conducted using Multiwfn(v3.8dev) [49]. The detailed parameters set are explained in the following paragraph.

Initial AIMD configurations were generated using Packmol [50], comprising five Na_2_WO_4_ units randomly placed within the simulation box, corresponding to a density of 3 g/cm^3^. The interatomic potential was evaluated at the DFT level using the van der Waals‐corrected PBE functional, consistent with previous AIMD studies of halide molten salt systems. The MOLOPT basis set with double‐zeta valence polarization (DZVP) quality was employed for all elements (Na, W, and O), and the electron density was expanded in reciprocal space with a plane‐wave cutoff of 350 Ry. AIMD simulations were conducted in the NVT ensemble at 1173 K, using a Nosé–Hoover thermostat to maintain temperature. To validate the generality of our conclusions, an additional Na_2_WO_4_–WO_3_ (3:1 ratio) and NaCl–KCl molten salt system containing 40 atoms (20 Cl, 14 K, and 6 Na) was simulated under the same computational conditions.

For deep potential modeling, we employed the DPA‐2.3.0‐v3.0.0b4‐medium [44] pretrained model from the DPA2 framework, which was trained across 28 diverse datasets to include the prerequisite general chemical knowledge, as shown in Figure 1. All other training hyperparameters were kept consistent with the original pretrained configuration, as detailed in the associated publication [44]. To evaluate the effectiveness of the pretrained model, this paper also employs a model with an identical architecture trained from scratch, which doesn't introduce any prerequisite knowledge. LAMMPS simulations were performed on systems containing 200 Na_2_WO_4_ units and Na_2_WO_4_–WO_3_ mixtures (3:1 ratio), both conducted in the NVT ensemble.

## 3 Experiment

### 3.1 Pre‐Trained Models and Models Trained from Scratch

#### 3.1.1 Predictive Performance

We evaluated the model's prediction accuracy by analyzing the Mean Absolute Error (MAE) and Standard Deviation (STD) on the validation set. The pretrained models for both the Na_2_WO_4_ and Na_2_WO_4_‐WO_3_ systems showed reductions in both metrics compared to models trained from scratch.Specifically, for Na_2_WO_4_, as shown in Table 1, the energy MAE decreased from 0.1378 to 0.0710 eV, and the force MAE was reduced from 0.0737 to 0.0556 eV/Å, representing significant improvements in prediction accuracy. Regarding the standard deviation analysis, a closer alignment between the standard deviation of the model's predictions and that of the DFT reference indicates that the model explores a broader feature space, providing insight into the degree of overfitting. The standard deviations from DFT calculations were 0.6703 eV for energy and 1.604 eV/Å for forces. As shown in Table 1, the error in the energy standard deviation (relative to the DFT reference) improved from 0.011 to ‐0.0016 eV after pretraining, an order‐of‐magnitude enhancement. The substantial reduction in standard deviation strongly suggests mitigated overfitting, indicating that the fine‐tuned model captures more genuine physical features rather than merely memorizing the training data. These results collectively confirm that, compared to training from scratch, pretraining produces a model with significantly enhanced accuracy and generalization capability.

**TABLE 1:** The performance of DPA2 and pre‐trained models on Na_2_WO_4_ and Na_2_WO_4_‐WO_3_ molten salt.

	DPA2‐from scratch		DPA2‐finetuned	
	Energy, eV	Force, eV/Å	Energy, eV	Force, eV/Å
MAE				
Na_2_WO_4_	0.1378	0.0737	0.0710	0.0556
Na_2_WO_4_‐WO_3_	0.0936	0.0740	0.0381	0.0557
STD				
Na_2_WO_4_	0.6813	1.605	0.6687	1.602
Na_2_WO_4_‐WO_3_	0.6068	1.381	0.6247	1.386

#### 3.1.2 Molecular Dynamics Simulation Performance

**FIGURE 2:**
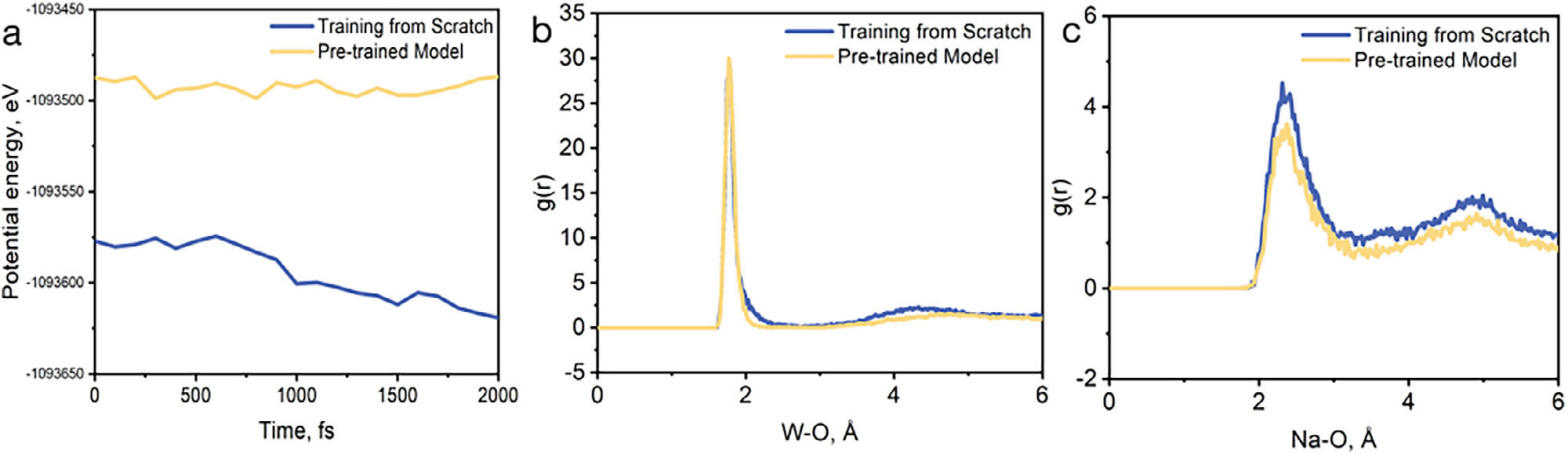
Comparison of molecular dynamics performance between pre‐trained and scratch‐trained models. (a)Energies output by different models during molecular dynamics simulations;(b) The radial distribution function (RDF)of W─O bonds in pre‐trained models and models trained from scratch;(c) The radial distribution function (RDF)of Na─O bonds in pre‐trained models and models trained from scratch.

Molecular dynamics (MD) simulations were performed on the Na_2_WO_4_‐WO_3_ mixture at 1173 K using the pretrained model. After geometry optimization, the system was equilibrated for 2000 fs in the NVT ensemble. We analyzed the evolution of potential energy and the W─O radial distribution function (RDF) throughout the trajectory. As shown in Figure 2a, the pretrained model yields more stable energy trajectories, with the system reaching equilibrium rapidly. In contrast, the from‐scratch model exhibits a slow downward drift, indicative of incomplete relaxation. A systematic offset of ∼100 eV is observed between the total energies of the two models, likely due to cumulative errors in atomic energy prediction. Figure 2b compares the W─O bond RDFs from both models. At short range (r <2 Å), the RDFs are nearly identical, indicating consistent descriptions of the primary W─O bonding environment. At longer distances, however, deviations emerge: the from‐scratch model produces broader peaks, reflecting its reduced accuracy in capturing medium‐range structural order.

These results collectively demonstrate the superior predictive accuracy and dynamic stability of the pretrained model. Accordingly, all subsequent simulations presented in this work are based on the pretrained model.

## 4 Discussion

In this section, we will use the trained model described above to investigate the underlying mechanisms of its energy predictions. We analyzed the outputs of the DPA2 model and identified a strong correlation between its atomic energy predictions and the integrated projected density of states (PDOS) within selected energy bands for each atom. To illustrate this point, we subsequently selected a random frame from both the model's training set and its validation set for detailed analysis. In parallel, we trained the model on a NaCl–KCl molten salt system and performed the same analysis to further validate the observed relationship.

**FIGURE 3:**
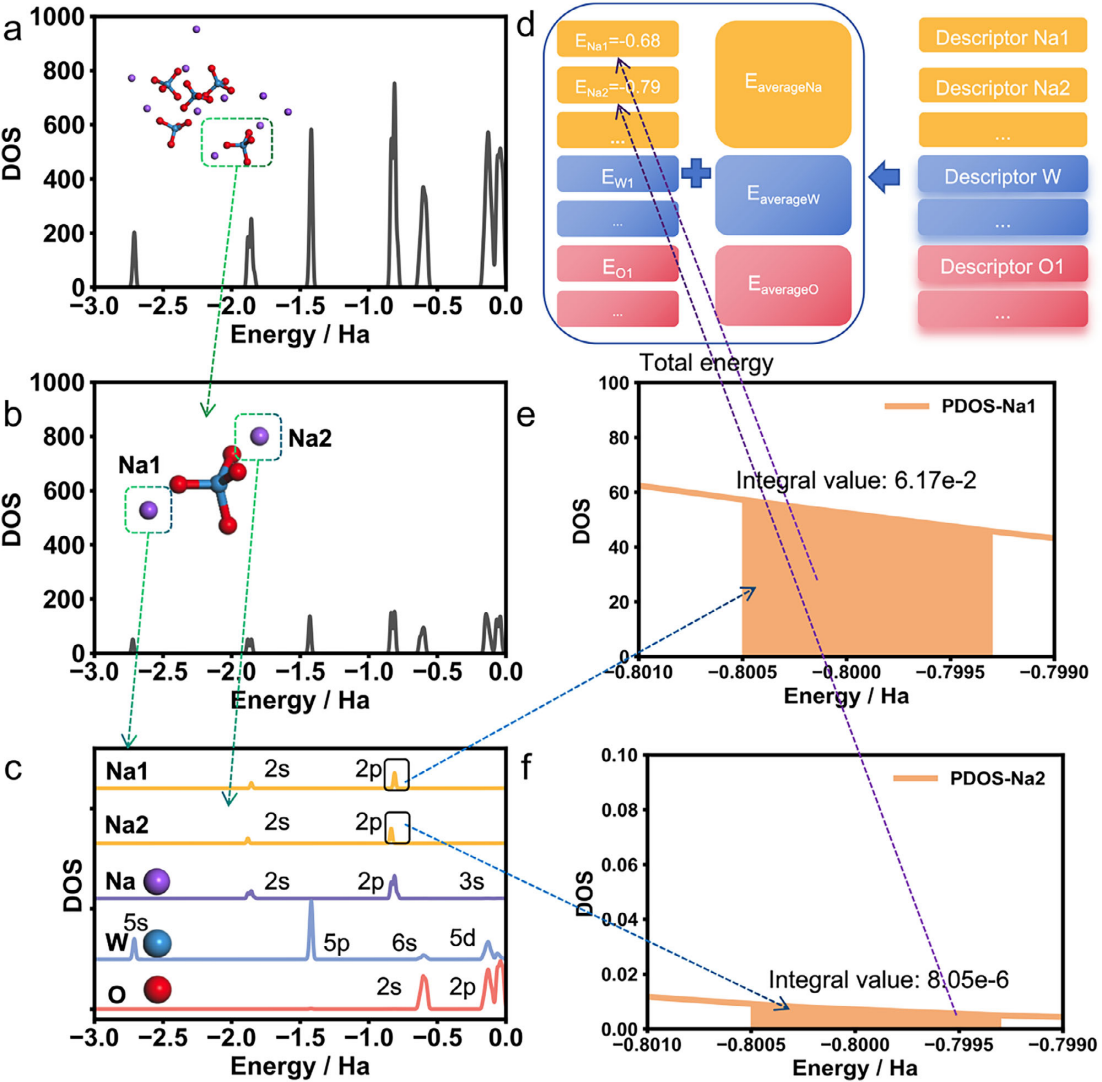
DOS of the Na_2_WO_4_ system and the workflow of the model.(a)TDOS of the Na_2_WO_4_ system;(b) PDOS of one Na_2_WO_4_; (c) PDOS of Na1 and Na2 in Na_2_WO_4_ and DOS of other atoms in vacuum; (d) Calculation process of the DPA model; (e) Enlarged integral range of Na1; (f) Enlarged integral range of Na2.

The DPA2 model computes the total energy through a multi‐stage process: atomic coordinates and element types are first embedded into descriptors via a neural network, which are then passed through a fitting network to map the energy contributions of individual atoms. Figure 3a shows the molecular structure of Na_2_WO_4_ used for subsequent analysis, with specific coordinate data and corresponding model‐predicted energies provided in Note S1. The total energy of the system is finally obtained by summing the energy contributions of all atoms. The energy of each atom again is the addition of two terms, that is, the average energy prior to training which originate from the statistically fitting of all training structures energy by the least‐squares method to determine an elemental reference energy for each atomic species, plus interaction energy, which are generated by the trained model by considering the environmental effects of each atom within. It should be noted that the atomic energies analyzed in this work exclude the average energy values of the respective elements because only the interaction energy are the interesting term representing the influence of the neighboring atoms and reflecting the physical properties of the system.

The density of states (DOS) provides a fundamental description of the local electronic environment from the perspective of molecular orbitals, essentially characterizing the distribution of available quantum states for electrons around a specific atom. To probe the element‐specific features and establish a direct link with the atomic contributions output by the machine learning model, we decomposed the total DOS obtained from Density Functional Theory (DFT) calculations into contributions from individual atoms (projected DOS, or PDOS). This atom‐resolved analysis allows us to directly compare the model's outputs with the underlying electronic structure, thereby examining whether the predicted atomic contributions possess a verifiable physical basis in the local chemical environment.

Our main results are summarized in Figure 3, where Figure 3a presents the total density of states (TDOS) of the Na_2_WO_4_ system corresponding to a randomly selected configuration from the training dataset of the DPA2 model. In this configuration, the basis sets include 9 valence electrons for Na, 14 for W, and 6 for O. As indicated by the green dashed arrows, we further analyzed the contributions of a single Na_2_WO_4_ molecule and individual atoms to the total TDOS. The projected density of states (PDOS) for the molecule and specific atoms was obtained by projecting the Kohn–Sham orbitals into the atomic basis functions corresponding to the target component (i.e., the selected molecule or atom), followed by summation of the contributions. Figure 3b,c shows the density of states (DOS) of one Na_2_WO_4_ molecule, the DOS of Na atoms in the two systems, and the DOS of Na, W, and O in vacuum, with the orbital character of each peak assigned accordingly. As shown by the blue dashed arrows, integration of the DOS curves over a specific energy band for each atom of the same element revealed a strong correlation between the resulting integral value and the model's output for that atom, as highlighted by the purple dashed arrows. Figure 3e,f illustrates the energy ranges used for the integration of Na1 and Na2 in this system.

The density of states (DOS) provides a fundamental description of the local electronic environment from the perspective of molecular orbitals, essentially characterizing the distribution of available quantum states for electrons around a specific atom. To probe the element‐specific features and establish a direct link with the atomic contributions output by the machine learning model, we decomposed the total DOS obtained from DFT calculations into contributions from individual atoms (projected DOS, or PDOS). This atom‐resolved analysis allows us to directly compare the model's outputs with the underlying electronic structure, thereby examining whether the predicted atomic contributions possess a verifiable physical basis in the local chemical environment.

**FIGURE 4:**
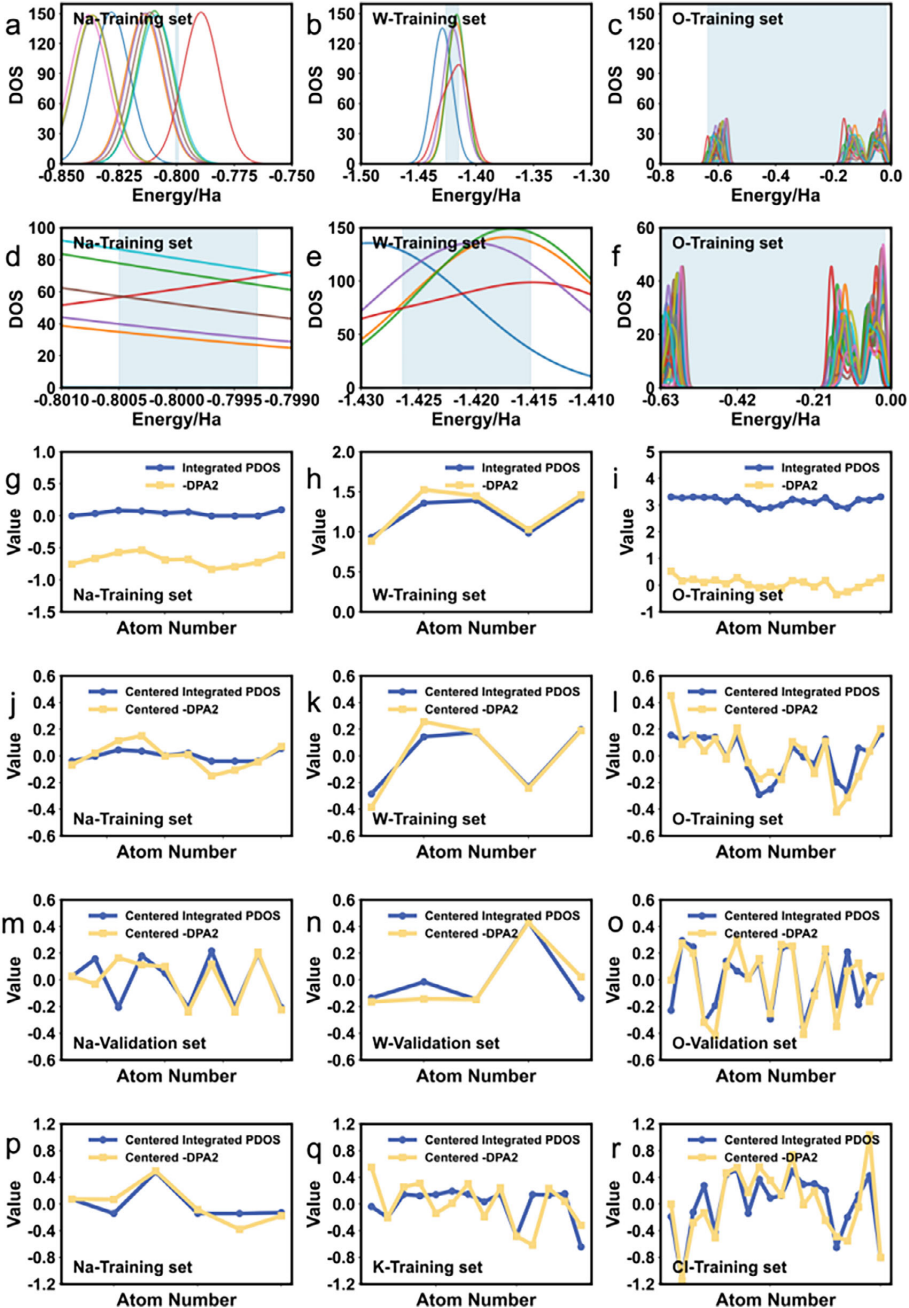
Comparison of PDOS integrals and model outputs for atoms in various systems.(a–c)PDOS and integration regions for atoms in the training set of the Na_2_WO_4_ model;(d–f)Enlarged views of the integration regions;(g–i)PDOS integrals and the negative of model outputs for each atom;(j–l)Centered results;(m–o)Validation set of the Na_2_WO_4_ model, centered PDOS integrals and model outputs for each atom;(p–r)Training set of the NaCl‐KCl model, centered PDOS integrals and model outputs for each atom.

Figure 4a–c highlights the integration regions corresponding to each element in this frame, while Figure 4d–f presents a magnified view of these regions. The energy ranges defining these integration regions were manually selected through an iterative process aimed at aligning the centered integrated PDOS values with the centered negative model outputs, thereby minimizing the discrepancy between these two quantities and reflecting the intended meaning of the model's predictions. Figure 4g–i shows the integrated values alongside the negative of the model outputs. The centered results of both quantities are shown in Figure 4j–l. We extended this analysis to both the validation set and a NaCl–KCl molten salt system, as illustrated in Figure 4m–r, where all systems exhibit good agreement. Specifically, Figure 4a displays the raw PDOS data for Na atoms from the training set, Figure 4d shows a magnified view of the integration range, Figure 4g presents the original integrated values alongside the negative values of the model outputs, and finally, Figure 4j illustrates the normalized results of these two datasets. After extensive investigation, we found that the integrated values near orbitals with relatively high DOS show remarkable agreement (after centering) with the negative of the model output, also after centering. For the validation set and the NaCl–KCl system, details on the integration ranges and corresponding model outputs are provided in Note S2–S5.

The observed correlation between the model‐predicted atomic energies and PDOS integrals over specific energy ranges can be rationalized by comparison of the real physical DFT energies of the system and the energy generated by the DPA2 model. As we already explained, DPA2 are trained to predict the interaction component—that is, the part of the energy that varies with atomic configuration and bonding. According to the molecular orbital theory, DFT energy is determined by the electron density of the entire electronic structure system, which are the combination of all molecular orbitals, of which, the frontier orbitals such as HOMO (highest occupied molecular orbital) and LUMO (lowest unoccupied molecular orbital), can influence the interactions and properties of the system. In this context, the PDOS provides a local projection of the molecular orbital structure. Critically, the integral of the PDOS over an energy window centered on the Fermi level effectively quantifies an atom's electron density in these frontier orbitals, which are the primary determinants of chemical bonding and reactivity. As shown in the blue area in Figure 4d–f, the model's success stems from its implicit focus on the subregions of these orbitals where the PDOS varies most significantly among atoms of the same species, rather than on full orbital features that are largely invariant. This allows the model to associate subtle changes in frontier electron density with variations in interaction energy. The observed strong correlation reveals a physically grounded mechanism: atoms with greater electron occupancy in bonding‐sensitive frontier states contribute more negatively (i.e., more stabilizingly) to the interaction energy. These findings suggest that the model, trained solely on atomic coordinates, has internalized a key principle of frontier orbital theory: the local occupancy of frontier orbitals dictates the interatomic interaction energy. Frontier orbital theory is the one of most important theories to understand the mechanism of chemical interactions, and our work demonstrated that the descriptors of model can indeed captured the fundamental properties of critical orbitals and build a direct relationship with the total energy of reaction. Thus, our interpretive framework is inherently general and can be successfully extended to any molecular or condensed‐phase systems where interatomic interactions are governed by the local electronic structure. This provides a robust strategy for building physically interpretable machine‐learned potentials across diverse chemical spaces.

In summary, this analysis provides detailed numerical evidence that the model learns physically meaningful representations when mapping atomic coordinates and element types to DFT interaction energies, rather than relying on statistical correlations or generating arbitrary outputs. The strong correspondence between model predictions and PDOS integrals over environment‐sensitive energy windows indicates that the model implicitly encodes key features of the electronic structure that govern interatomic interactions—even though it is trained solely on structural and energetic data. The established correlation opens novel avenues for model refinement through strategies such as incorporating electronic structure features—especially those sensitive to the local chemical environment—as supervisory signals, which could offer a more physically grounded learning objective than total energy alone. Ultimately, by linking the model's predicted atomic interactions to PDOS‐derived quantities, this work deepens our understanding of how neural networks can reconstruct DFT‐level energies from atomic geometry. This understanding paves the way for developing more accurate models in the future, which could ultimately provide reliable theoretical principles and predictive guidance for experimental design and analysis.

## 5 Conclusion

We fine‐tuned a pretrained deep potential model using ab initio molecular dynamics (AIMD) data of molten salt, and through detailed analysis of the model outputs, we found that it encodes physically meaningful information beyond simple energy prediction. During our study of decomposing the system's total density of states (TDOS) into atomic projected density of states (PDOS), we uncovered a close correlation between the model‐predicted atomic energy contributions and the number of atom‐associated electronic states, specifically in energy bands featuring high electron density and distinct local environments. Our findings confirm that the primary physical mechanism behind MLP models lies in its descriptors, which map local atomic environments onto atomic contributions that implicitly encode the underlying electronic structure. Because the local atomic environment directly dictates the characteristics and occupancy of the frontier orbitals, these orbitals represent the fundamental physical information that determines the final model outputs. This ensures that the model's predictions reflect intrinsic physico‐chemical interactions derived from the local environment rather than mere statistical patterns. These findings highlight the physical interpretability of deep potential models and establish a practical framework for linking machine‐learned atomic energies to quantum mechanical descriptors such as PDOS. This not only offers a new route for model evaluation and improvement but also provides insights into atomic‐scale interactions in complex condensed‐phase systems. Finally, our study confirms the applicability of the fine‐tuned model to molten salt systems and demonstrates its superior accuracy and stability compared to a counterpart trained from scratch using the same architecture.

## Author Contributions

Y.L.G. conceived the idea and designed the work. Y.L.G. performed the experiments and wrote the manuscript. X.B.S. supervised the research, discussed the results, and reviewed the manuscript. X.L.X. and Z.R.N. acquired funding, provided resources, and supervised the project.

## Conflicts of Interest

The authors declare no conflicts of interest.

## Supporting information




**Supporting file**: advs74291‐sup‐0001‐SuppMat.docx

## Data Availability

The data that support the findings of this study are available in the supplementary material of this article.
