# Supplementary material for: Mechanistic Analysis of Large Atomic Models of Molten Salt
Source: Adv Sci (Weinh). 2026 Mar 10;13(22):e22313. doi: 10.1002/advs.202522313 (PMC13088276; doi:10.1002/advs.202522313)
Supplement: Supplementary file 1 — Supporting file: advs74291‐sup‐0001‐SuppMat.docx [file ADVS-13-e22313-s001.docx]

Mechanistic Analysis of Pretrained Large Atomic Models in Molten Salt

Supplementary Information

Yuliang Guo, Xiaobo Sun, Xiaoli Xi*, Zuoren Nie

**Supplementary Note 1. One Na_2_WO_4_ data from the training set and model predictions.**

**Supplementary Table 1:** One Na_2_WO_4_ data from the training set and model predictions. The PDOS integrals were computed using Multiwfn software based on wavefunction files generated by CP2K. Specifically, the integration ranges were set to -0.8005 to -0.7993 Hartree for Na, -1.4264 to -1.4153 Hartree for W, and -0.6362 to -0.0173 Hartree for O.

| No. | Element | X | Y | Z | Integrated PDOS | Model Output |
| --- | --- | --- | --- | --- | --- | --- |
| 1 | Na | 8.0126 | 4.3784 | 9.0285 | 0.0003 | 0.7538 |
| 2 | Na | -0.8635 | 5.2916 | 3.0013 | 0.0368 | 0.6641 |
| 3 | Na | 4.1985 | 11.3378 | 3.3530 | 0.0852 | 0.5718 |
| 4 | Na | 3.9904 | 5.1490 | 4.5232 | 0.0752 | 0.5344 |
| 5 | Na | 5.2007 | 15.9388 | -1.7239 | 0.0422 | 0.6868 |
| 6 | Na | 10.3569 | 12.5283 | 5.5785 | 0.0617 | 0.6773 |
| 7 | Na | 6.4335 | 0.8402 | 8.7983 | 5.68e-6 | 0.8344 |
| 8 | Na | 11.3895 | 9.8232 | -1.4196 | 8.05e-6 | 0.7929 |
| 9 | Na | 2.4524 | 15.1047 | 0.6575 | 7.29e-6 | 0.7297 |
| 10 | Na | 7.3506 | 8.3663 | 5.1283 | 0.0957 | 0.6146 |
| 11 | W | 10.9080 | 12.0945 | 1.8802 | 0.9326 | -0.8865 |
| 12 | O | 9.5491 | 13.2583 | 1.5171 | 3.3094 | -0.5239 |
| 13 | O | 12.5389 | 13.0141 | 2.0337 | 3.2741 | -0.1577 |
| 14 | O | 10.6918 | 11.4041 | 3.4639 | 3.3077 | -0.2266 |
| 15 | O | 10.7373 | 10.8959 | 0.5188 | 3.2911 | -0.1078 |
| 16 | W | 6.7085 | 3.5290 | 6.0263 | 1.3602 | -1.5273 |
| 17 | O | 6.9915 | 1.9074 | 6.6451 | 3.2952 | -0.1984 |
| 18 | O | 5.6034 | 3.4461 | 4.6061 | 3.1474 | -0.0496 |
| 19 | O | 5.9445 | 4.6427 | 7.1730 | 3.3063 | -0.2814 |
| 20 | O | 8.2239 | 4.2774 | 5.2800 | 3.0664 | -0.0206 |
| 21 | W | 5.7278 | 7.3318 | 1.9502 | 1.3935 | -1.4505 |
| 22 | O | 6.6852 | 6.4820 | 0.6245 | 2.8640 | 0.1013 |
| 23 | O | 6.9652 | 7.0261 | 3.3507 | 2.9034 | 0.0507 |
| 24 | O | 5.7643 | 9.0468 | 1.3327 | 3.0099 | 0.1054 |
| 25 | O | 4.1847 | 6.4372 | 2.1127 | 3.2196 | -0.1773 |
| 26 | W | 4.5547 | 8.9892 | 5.5790 | 0.9849 | -1.0295 |
| 27 | O | 5.4206 | 9.4518 | 4.1475 | 3.1462 | -0.1210 |
| 28 | O | 3.9417 | 7.4578 | 5.6775 | 3.0935 | 0.0605 |
| 29 | O | 5.7292 | 8.7844 | 6.9186 | 3.2800 | -0.1795 |
| 30 | O | 3.3838 | 10.4655 | 5.9167 | 2.9583 | 0.3478 |
| 31 | W | 10.5198 | 6.2141 | 6.8210 | 1.4135 | -1.4606 |
| 32 | O | 11.6241 | 5.0110 | 5.7949 | 2.8911 | 0.2411 |
| 33 | O | 9.5415 | 7.1794 | 5.7258 | 3.2123 | 0.0837 |
| 34 | O | 9.7538 | 5.0499 | 7.9647 | 3.1864 | -0.1067 |
| 35 | O | 11.4776 | 7.2117 | 7.9393 | 3.3170 | -0.2738 |

**Supplementary Note 2. One Na_2_WO_4_ data from the validation set and model predictions.**

**Supplementary Table 2:** One Na_2_WO_4_ data from the validation set and model predictions. The PDOS integrals were computed using Multiwfn software based on wavefunction files generated by CP2K. Specifically, the integration ranges were set to -0.8186 to -0.8141 Hartree for Na, -1.3999 to -1.3932 Hartree for W, and -0.6355 to -0.6049 Hartree for O.

| No. | Element | X | Y | Z | Integrated PDOS | Model Output |
| --- | --- | --- | --- | --- | --- | --- |
| 1 | Na | 6.3959 | 6.3308 | 9.6792 | 0.2359 | 0.7134 |
| 2 | Na | 0.8045 | 1.6844 | 5.2702 | 0.3662 | 0.7724 |
| 3 | Na | 3.4113 | 4.7386 | 4.5462 | 0.0024 | 0.5750 |
| 4 | Na | 3.3265 | 5.6421 | 8.3123 | 0.3892 | 0.6279 |
| 5 | Na | 2.9241 | 9.6668 | 0.2131 | 0.2594 | 0.6403 |
| 6 | Na | 9.0651 | 7.9403 | 7.2215 | 0.0002 | 0.9803 |
| 7 | Na | 6.3925 | 1.5793 | 8.5689 | 0.4258 | 0.6223 |
| 8 | Na | 10.2387 | 6.1251 | 1.0876 | 4.64e-5 | 0.9804 |
| 9 | Na | 5.0845 | 10.5877 | 3.6125 | 0.4067 | 0.5325 |
| 10 | Na | 6.8974 | 3.9802 | 5.4305 | 0.0007 | 0.9646 |
| 11 | W | 8.3053 | 8.5782 | 1.7726 | 0.0121 | -1.2399 |
| 12 | O | 10.0822 | 9.0125 | 1.5089 | 0.1439 | -0.0971 |
| 13 | O | 7.4580 | 8.7438 | 0.1988 | 0.6677 | -0.3727 |
| 14 | O | 8.0671 | 6.8647 | 2.2195 | 0.6209 | -0.2991 |
| 15 | O | 7.3706 | 9.6747 | 2.9075 | 0.0543 | 0.2203 |
| 16 | W | 4.4138 | 2.7653 | 6.4479 | 0.1331 | -1.2615 |
| 17 | O | 3.8033 | 1.9084 | 7.8321 | 0.1807 | 0.3199 |
| 18 | O | 4.6131 | 4.4854 | 6.6434 | 0.5130 | -0.2002 |
| 19 | O | 6.0088 | 2.0440 | 6.0415 | 0.4390 | -0.3990 |
| 20 | O | 3.1409 | 2.4759 | 5.2835 | 0.3849 | -0.1067 |
| 21 | W | 4.2066 | 3.8355 | 1.7300 | 0.0016 | -1.2567 |
| 22 | O | 5.0454 | 4.2978 | 3.2615 | 0.5058 | -0.2573 |
| 23 | O | 2.5660 | 4.5563 | 2.1211 | 0.0797 | 0.1549 |
| 24 | O | 4.9155 | 4.6876 | 0.3241 | 0.6088 | -0.3633 |
| 25 | O | 4.2756 | 2.1023 | 1.5742 | 0.6262 | -0.3501 |
| 26 | W | 3.0204 | 8.1510 | 6.1042 | 0.5818 | -1.8328 |
| 27 | O | 3.9850 | 8.1720 | 7.7005 | 0.0224 | 0.3100 |
| 28 | O | 2.5199 | 6.5302 | 5.8542 | 0.2854 | 0.0200 |
| 29 | O | 1.5399 | 8.9999 | 6.4461 | 0.5655 | -0.3291 |
| 30 | O | 3.9783 | 8.7172 | 4.8191 | 0.1880 | 0.2517 |
| 31 | W | 9.3353 | 4.1123 | 7.8666 | 0.0120 | -1.4270 |
| 32 | O | 9.6412 | 3.5644 | 6.2096 | 0.5835 | -0.1641 |
| 33 | O | 8.2928 | 5.6595 | 7.7480 | 0.1881 | -0.2229 |
| 34 | O | 8.2320 | 2.9936 | 8.6366 | 0.4044 | 0.0640 |
| 35 | O | 10.9102 | 4.1824 | 8.7208 | 0.3924 | -0.1239 |

**Supplementary Note 3. One NaCl-KCl data from the training set and model predictions.**

**Supplementary Table 3:** One NaCl-KCl data from the training set and model predictions. The PDOS integrals were computed using Multiwfn software based on wavefunction files generated by CP2K. Specifically, the integration ranges were set to -0.9024 to -0.8520 Hartree for Na, -0.5977 to -0.4611 Hartree for K, and -0.4506 to -0.0264 Hartree for Cl.

| No. | Element | X | Y | Z | Integrated PDOS | Model Output |
| --- | --- | --- | --- | --- | --- | --- |
| 1 | Na | 9.4315 | 2.7150 | 2.1692 | 0.2145 | 0.6863 |
| 2 | Na | 10.0038 | 6.6317 | 8.0489 | 8.0e-8 | 0.6906 |
| 3 | Na | 13.3559 | 4.0109 | 3.3081 | 0.6222 | 0.2655 |
| 4 | Na | 5.7406 | 9.2640 | 5.4659 | 8.0e-8 | 0.8442 |
| 5 | Na | 4.8880 | 1.0356 | 1.5732 | 1.7e-5 | 1.1393 |
| 6 | Na | 2.6650 | 7.1353 | 0.2897 | 0.0135 | 0.9379 |
| 7 | K | 13.5098 | 10.5756 | 9.0276 | 2.8226 | 0.1333 |
| 8 | K | 5.4726 | 7.7889 | 11.0808 | 2.6592 | 0.8895 |
| 9 | K | 3.6205 | 4.7454 | 8.8360 | 3.0045 | 0.4298 |
| 10 | K | 11.3101 | 7.6804 | 1.2139 | 2.9834 | 0.3738 |
| 11 | K | 6.5961 | 3.7199 | 2.2625 | 3.0030 | 0.8237 |
| 12 | K | 1.8503 | 7.1771 | 9.4566 | 3.0549 | 0.6747 |
| 13 | K | 10.1812 | 10.0081 | 6.0527 | 3.0074 | 0.3795 |
| 14 | K | 4.3293 | 8.3056 | 2.7551 | 2.8958 | 0.8744 |
| 15 | K | 0.5760 | 2.2622 | 0.2926 | 3.0095 | 0.4411 |
| 16 | K | 7.8812 | 1.0029 | 0.0336 | 2.3766 | 1.1671 |
| 17 | K | 8.8969 | 4.4280 | 8.7313 | 3.0032 | 1.2988 |
| 18 | K | 0.3514 | 9.2148 | 4.0155 | 2.9945 | 0.4502 |
| 19 | K | 5.8385 | 10.4409 | 8.4091 | 3.0151 | 0.6443 |
| 20 | K | 9.0272 | 9.4198 | 2.2737 | 2.2189 | 1.0039 |
| 21 | Cl | 2.4402 | 8.4270 | 6.0771 | 2.2642 | 0.0283 |
| 22 | Cl | 4.9975 | 4.9858 | 5.9192 | 1.3025 | 1.1803 |
| 23 | Cl | 11.1350 | 6.4606 | 10.7920 | 2.3295 | 0.3052 |
| 24 | Cl | 9.1943 | 2.4933 | 6.1292 | 2.7301 | 0.1588 |
| 25 | Cl | 3.3628 | 11.9507 | 8.2969 | 2.0377 | 0.5331 |
| 26 | Cl | 5.0852 | 4.9275 | -0.0022 | 2.8918 | -0.4418 |
| 27 | Cl | 1.6643 | 9.6141 | 11.1144 | 2.9653 | -0.5212 |
| 28 | Cl | 11.6143 | 6.6952 | 4.6127 | 2.3140 | -0.1429 |
| 29 | Cl | 6.7252 | 4.8203 | 11.1054 | 2.8238 | -0.5301 |
| 30 | Cl | 11.6593 | 4.5279 | 1.4292 | 2.5425 | -0.3267 |
| 31 | Cl | 8.7525 | 5.5284 | 1.5972 | 2.5783 | -0.1077 |
| 32 | Cl | 11.2897 | 0.9634 | 2.9273 | 2.9432 | -0.7110 |
| 33 | Cl | 5.0592 | 7.3728 | 8.0265 | 2.7473 | 0.0364 |
| 34 | Cl | 2.4706 | 2.5141 | 3.0540 | 2.7569 | -0.1683 |
| 35 | Cl | 4.0508 | 3.0328 | 11.0807 | 2.6542 | 0.2692 |
| 36 | Cl | 2.4972 | 5.5657 | 4.1760 | 1.7998 | 0.5135 |
| 37 | Cl | 11.4608 | 9.5719 | 11.0914 | 2.2559 | 0.5818 |
| 38 | Cl | 8.2850 | 6.9176 | 5.2613 | 2.5862 | 0.0734 |
| 39 | Cl | 7.2810 | 0.9188 | 3.5559 | 2.8785 | -1.0113 |
| 40 | Cl | 0.1595 | 2.3075 | 7.2384 | 1.6491 | 0.8289 |

**Supplementary Note 4. Integration range for the Na_2_WO_4_ system selected from the validation set.**

**
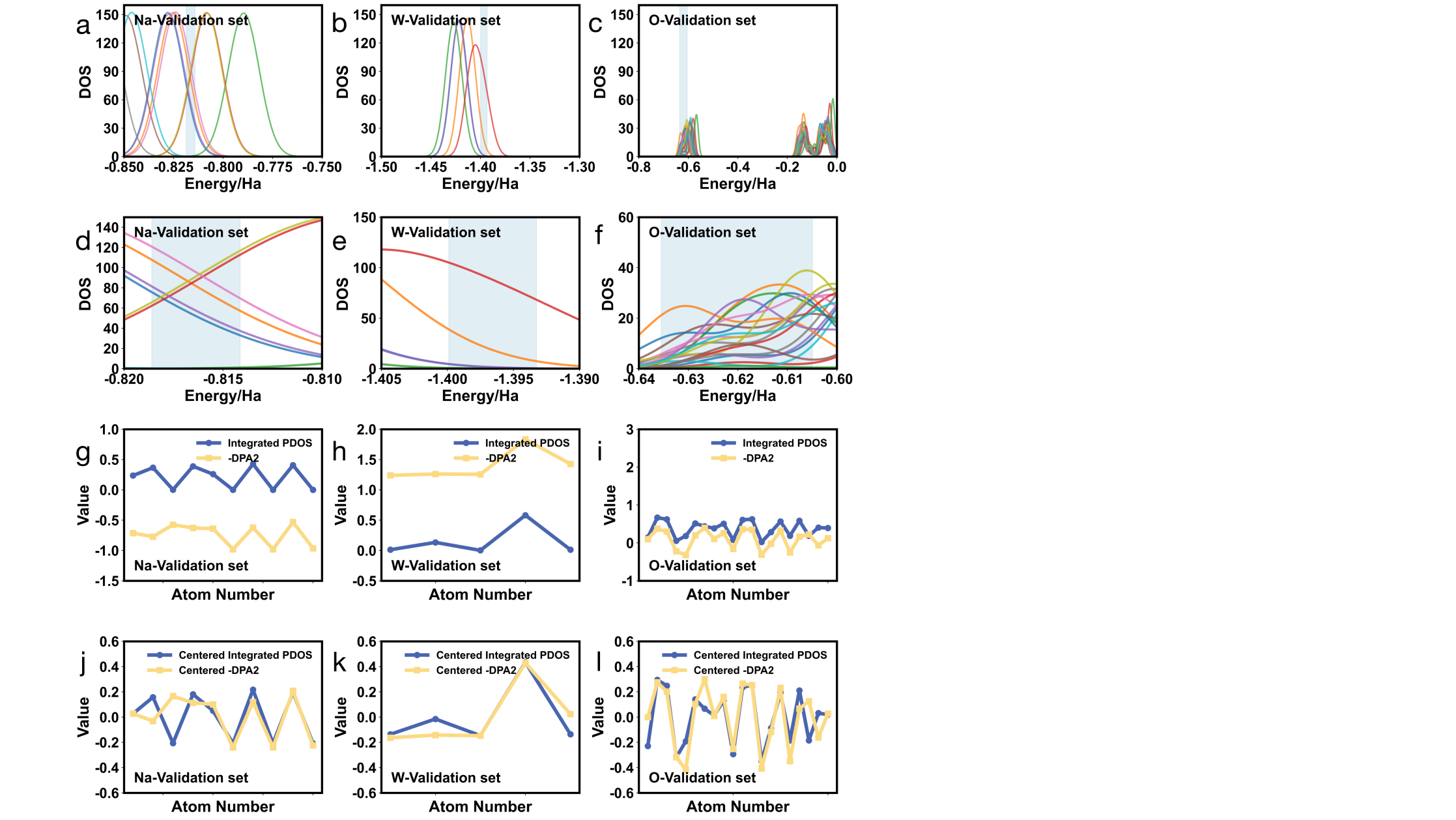
**

**Supplementary Figure 1:** Comparison of PDOS integrals and model outputs for atoms in Na_2_WO_4_ system selected from the validation set. (a-c)PDOS and integration regions for atoms; (d-f)Enlarged views of the integration regions; (g-i)PDOS integrals and the negative of model outputs for each atom; (j-l)Centered results.

**Supplementary Note 5. Integration range for the NaCl-KCl system selected from the training set.**

**
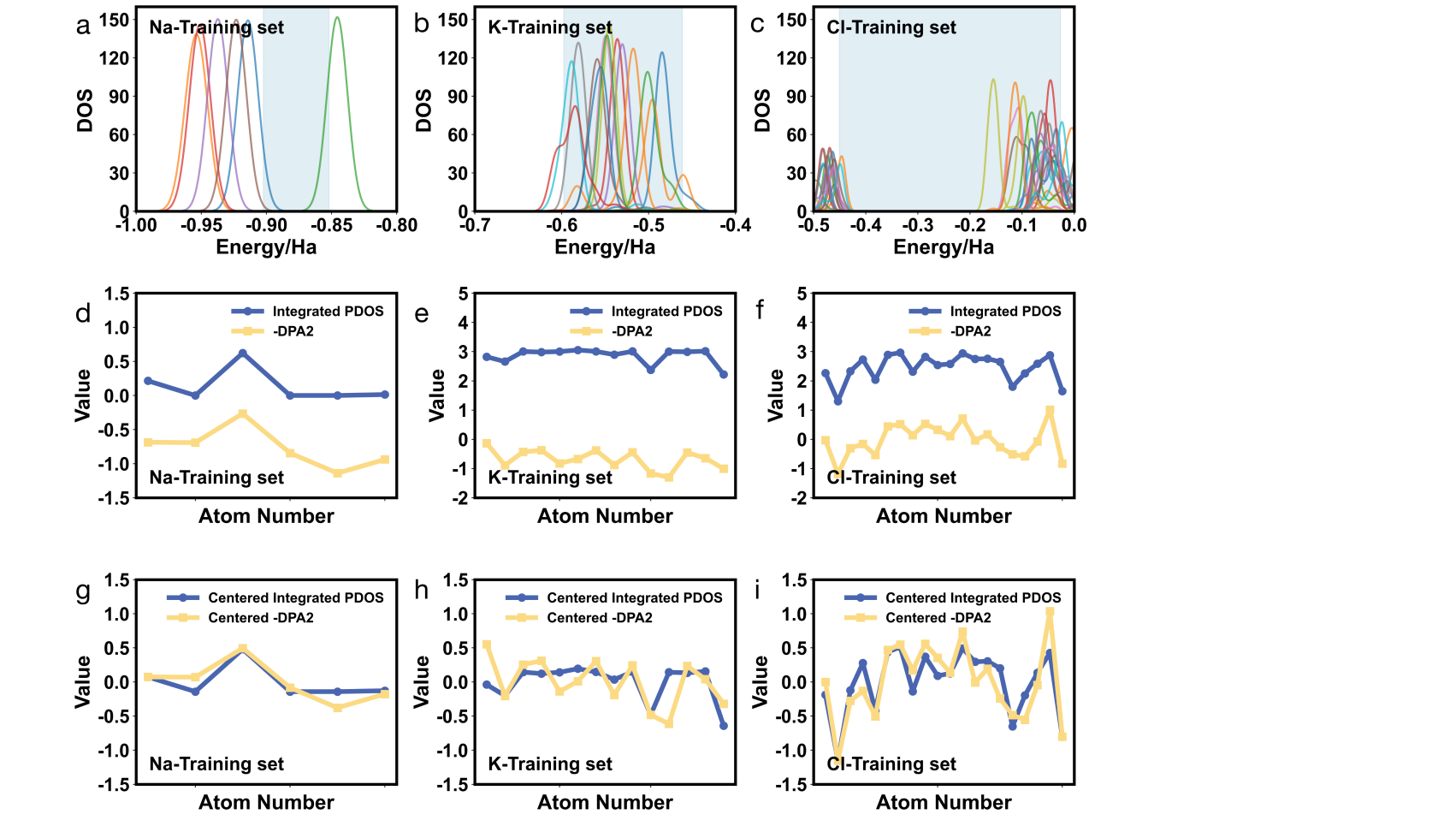
**

**Supplementary Figure 2:** Comparison of PDOS integrals and model outputs for atoms in NaCl-KCl system selected from the validation set. (a-c)PDOS and integration regions for atoms; (d-f)PDOS integrals and the negative of model outputs for each atom; (g-i)Centered results.
